# Geobotanical Study, DNA Barcoding, and Simple Sequence Repeat (SSR) Marker Analysis to Determine the Population Structure and Genetic Diversity of Rare and Endangered *Prunus armeniaca* L.

**DOI:** 10.3390/plants14152333

**Published:** 2025-07-28

**Authors:** Natalya V. Romadanova, Nazira A. Altayeva, Alina S. Zemtsova, Natalya A. Artimovich, Alexandr B. Shevtsov, Almagul Kakimzhanova, Aidana Nurtaza, Arman B. Tolegen, Svetlana V. Kushnarenko, Jean Carlos Bettoni

**Affiliations:** 1 Institute of Plant Biology and Biotechnology, 45 Timiryazev St., Almaty 050040, Kazakhstan; zemtsovaaalina@gmail.com (A.S.Z.); artimovich_nata@mail.ru (N.A.A.); tolegenarman7@gmail.com (A.B.T.); sv.kushnarenko.bio@gmail.com (S.V.K.); 2 National Center for Biotechnology, 13/5, Kurgalzhynskoye Road, Astana 010000, Kazakhstan; ncbshevtsov@gmail.com (A.B.S.); kakimzhanova@mail.ru (A.K.); aid306@mail.ru (A.N.); 3 Faculty of Biology and Biotechnology, Al-Farabi Kazakh National University, Al-Farabi Av. 71, Almaty 050040, Kazakhstan; 4 The New Zealand Institute for Plant and Food Research Limited, Canterbury Agriculture & Science Centre, 74 Gerald St, Lincoln 7608, New Zealand

**Keywords:** *Rosaceae*, endangered species, plant descriptors, SSR markers, *Prunus* spp.

## Abstract

The ongoing genetic erosion of natural *Prunus armeniaca* populations in their native habitats underscores the urgent need for targeted conservation and restoration strategies. This study provides the first comprehensive characterization of *P. armeniaca* populations in the Almaty region of Kazakhstan, integrating morphological descriptors (46 parameters), molecular markers, geobotanical, and remote sensing analyses. Geobotanical and remote sensing analyses enhanced understanding of accession distribution, geological features, and ecosystem health across sites, while also revealing their vulnerability to various biotic and abiotic threats. Of 111 morphologically classified accessions, 54 were analyzed with 13 simple sequence repeat (SSR) markers and four DNA barcoding regions. Our findings demonstrate the necessity of integrated morphological and molecular analyses to differentiate closely related accessions. Genetic analysis identified 11 distinct populations with high heterozygosity and substantial genetic variability. Eight populations exhibited 100% polymorphism, indicating their potential as sources of adaptive genetic diversity. Cluster analysis grouped populations into three geographic clusters, suggesting limited gene flow across Gorges (features of a mountainous landscape) and greater connectivity within them. These findings underscore the need for site-specific conservation strategies, especially for genetically distinct, isolated populations with unique allelic profiles. This study provides a valuable foundation for prioritizing conservation targets, confirming genetic redundancies, and preserving genetic uniqueness to enhance the efficiency and effectiveness of the future conservation and use of *P. armeniaca* genetic resources in the region.

## 1. Introduction

*Prunus armeniaca* L., commonly known as apricot, is widely grown around the world [1]. Wild apricot populations thrive in the mountains of Central Asia and China, from Kashmir to Tien-Shan, suggesting that these regions, including Kazakhstan, Kyrgyzstan, Tajikistan, Southern China, and Northern India, are believed to be the center of origin for domesticated apricot varieties [2,3,4,5]. As whole plants grow in situ, apricots have a limited life span, usually determined by the aging of the plants, weather conditions, and the threat of diseases and pests. Anthropogenic activities and natural climatic changes have severely impacted these populations, contributing to their endangered status and inclusion in the Red Book of Kazakhstan [6]. *P. armeniaca* L., a highly polymorphic species, exhibits significant genetic and phenotypic diversity within populations, with trees varying in crown shape, density, leaf morphology, and fruit size, shape, color, and flavor [4,7,8,9,10,11]. Wild populations in the Trans-Ili Alatau and Dzungarian Alatau mountains of Kazakhstan demonstrate remarkable resilience and adaptability, tolerating winter temperatures as low as −30 °C and showing robust disease resistance [11]. Beyond ecological roles, this biodiversity holds economic value as a genetic resource for future needs [8,11,12]. Wild Kazakhstani *P. armeniaca* fruits have a nutritional profile comparable to or surpassing European cultivated varieties, with similar sugar (7.6–11.5%) and fiber (0.96%) content, and higher organic acids (2.35%) and pectin (1.85%) [8,13,14].

The loss of genetic diversity in wild populations can lead to significant ecological damage and potentially irreversible consequences for the population’s ability to adapt and survive future challenges. Plant biodiversity has been significantly affected, directly or indirectly, by human activities, which have led to a decline in native habitats, resulting not only in the loss of plant species biomass, but also in their diversity [15,16,17,18,19]. According to Bar-On et al. [18], approximately 40% of the planet’s total biomass (around 220 gigatons of carbon biomass) has been lost in the last few thousand years. Recent estimates by the Global Tree Assessment, coordinated by the Botanic Gardens Conservation International and the International Union for Conservation of Nature, have identified that a substantial 30% of tree species are threatened with extinction, with at least 142 tree species having already been recorded as extinct in the wild [20]. The risk of losing many valuable plant genetic resources, including those that are subject to this study, underscores the urgency of developing integrated efforts to identify and conserve these resources for future use [21,22,23,24,25,26].

Understanding geographic distribution, population dynamics, and ecological interactions is critical for assessing resilience and prioritizing conservation [27,28,29,30]. Geobotanical studies, including morphological assessments during field expeditions, provide essential data on plant health and community diversity [31,32,33,34]. This set of data enables botanists to accurately identify a plant species by comparing their observations with known species descriptions [34,35,36,37]. Regardless of whether they are based on qualitative or quantitative traits, species descriptions are usually developed by botanists or taxonomists. These descriptions may involve international organizations, such as the International Plant Genetic Resources Institute (IPGRI-Bioversity International) [38], the European Cooperative Programme for Plant Genetic Resources (ECPGR) [32,39], and the Food and Agriculture Organization of the United Nations (FAO) [31].

Geographic Information Systems (GIS) technologies, including remote sensing, complement standardized field expeditions to create geographic maps of plant growth sites and understand their spatial distribution within a given area [40,41,42,43,44,45]. GIS technologies make it possible to integrate large amounts of cartographic (maps) and thematic (data related to these maps) information into a single, coordinated structure. This includes remote sensing data, field research, and—equally importantly—an interdisciplinary and transdisciplinary knowledge team. This team includes sensing experts, botanists, and conservation scientists [46,47,48,49,50,51,52,53].

Accurately characterizing genetic diversity within and among populations is essential for effective conservation efforts. This enables experts to identify redundancies, confirm uniqueness, and increase conservation efficiency [54,55]. The polymorphic nature and phenotypic plasticity of *Prunus* spp. pose significant challenges to traditional field identification based solely on physical traits [9,10,11,56]. In this sense, molecular markers, such as DNA barcoding and microsatellites, have become a very powerful tool for studying the genetic diversity and population structure [56,57,58]. They provide a more accurate and rapid way to identify plant species, especially when differentiation based on morphological traits is unreliable or unavailable and phenotypic plasticity is a concern. Rather than relying solely on physical characteristics, species identification is based on genetic sequence comparison [59,60]. DNA barcoding and microsatellites are well-established and widely used methods for species identification in many taxa [60,61,62,63]. They can also be used alongside traditional morphological methods to characterize a new plant species [64,65,66]. DNA barcoding uses standardized DNA sequences (barcodes) to identify species by comparing them to a database. Microsatellites, on the other hand, are highly variable genetic markers due to their high degree of polymorphism. This variability makes them valuable for studying genetic diversity, especially when DNA barcodes fail to differentiate species [67,68]. Although DNA barcoding is effective, it can fail, particularly when the barcode gap is insufficient [68]. In other words, DNA barcoding relies on the barcode gap to reliably distinguish species based on their DNA sequences. However, when this gap is small or absent, distinguishing species becomes difficult or impossible, which can lead to misidentification [61,69]. DNA barcoding and microsatellites can also be used together as complementary approaches to identify and differentiate species, especially closely related ones [68]. DNA barcoding can serve as the primary identification tool, and microsatellites can be used to distinguish closely related species more precisely when barcoding alone is insufficient.

In plant science, DNA-level research is essential for many applications, including plant breeding, taxonomy, biodiversity assessment, conservation, and genetics/genomics studies [70,71,72,73,74,75]. Besides the molecular characterization of the plant genetic resources for genetic diversity, DNA-level research enables scientists to identify specific genetic variations, or polymorphisms, and to understand the molecular mechanisms underlying phenotypic variability [76,77]. Furthermore, it allows scientists to identify and characterize genes associated with specific traits [77]. The genetic diversity of *Prunus* species has been extensively studied around the globe using molecular markers, such as simple sequence repeat (SSR) markers. As a result, many SSR markers have been developed for different *Prunus* species, including apricots and their wild relatives [57,78,79,80,81,82,83,84]. However, studies on the genetic diversity of apricots in Kazakhstan, particularly those using techniques such as SSR and DNA barcoding, are still relatively limited, despite their importance in a likely center of origin [85]. Obtaining this information is crucial for a better understanding of the genetic diversity and structure of apricot populations in Kazakhstan. It would allow for more targeted conservation strategies and improved breeding programs. This study characterizes *P. armeniaca* populations in the Almaty region for the first time using international morphological descriptors (46 parameters) and molecular markers. Growth pattern maps were compiled, and genetic diversity and population structure were analyzed using 13 simple sequence repeat (SSR) markers and four different DNA barcoding regions. The current study establishes a scientific foundation for the efficient conservation and use of available *P. armeniaca* genetic resources. Additionally, the results will further support breeding programs for crop improvement.

## 2. Results and Discussion

### 2.1. Description of Populations

Central Asia, a major center of origin and diversity for fruit tree species, including apricots, has experienced a significant decline in natural apricot populations over the years. The presence of wild apricot forests in the mountainous regions of Kazakhstan during the mid-20th century is well documented. Botanists who explored these areas recorded naturally occurring stands of densely growing wild apricots that coexisted with other species of wild fruit trees [3,86,87,88,89,90]. However, the current state of these populations reflects a significant decline, driven primarily by sustained anthropogenic pressures and the effects of climate change. As a result, wild apricot trees now persist primarily as sparse stands, small groups, or isolated individuals within their remaining natural habitats [90,91,92]. The ongoing genetic erosion of natural apricot populations emphasizes priority of measures aimed at conservation and restoration of the genetic structure of these populations [34,91].

The *P. armeniaca* populations that were the focus of this study were found primarily growing on mountain slopes and in foothills at altitudes ranging from 862 to 1692 m above sea level. Field observations conducted during the expeditions revealed the recurring impact of climatic events, particularly spring frosts coinciding with the flowering stage of wild apricot trees. The spring of 2024 was the 4th consecutive year in which frost adversely affected flowering, thereby disrupting fruit development. These repeated climatic events have significantly disrupted the reproductive cycle of wild apricot populations, particularly by inhibiting seed production. The trees encountered during fieldwork were mostly adults, and their fruiting patterns varied widely across populations, ranging from a complete absence of fruit to a relatively abundant set, depending on local environmental conditions. All plant accessions that provided material during the expeditions were characterized and numbered. A total of 111 accessions were initially classified by botanists into two populations, which were subsequently characterized using standardized morphological descriptors. The characterization of these populations and their surrounding species is presented below. The distribution of key qualitative morphological traits for the two *P. armeniaca* populations is shown in Supplementary Table S1.

In the first population, 23 accessions of *P. armeniaca* were identified in Turgen Gorge (Enbekshikazakh District). These were primarily viable, non-fruiting, evergreen adult trees, most of which exceeded 8 m in height. The incidence of disease and pest infestation was low, with rust diseases (*Puccinia* spp.), *Cydia pomonella* (codling moth), and *Myzus mumecola* (apricot aphid) as the primary issues. A significant number of dry branches were also observed on older trees, possibly as a result of natural aging processes. The predominant plant species surrounding the collection site included *Berberis heteropoda* Schrenk, *Crataegus dsungarica* Zabel ex Lange, *Elaeagnus angustifolia* L., *Rosa platyacantha* Schrenk, and *Spiraea hypericifolia* L.

The second *P. armeniaca* population comprised 88 accessions collected from four distinct sites. At site 1, located in Bolshoy Aksu Gorge (Uyghur District), 43 accessions were identified. These accessions were predominantly viable, non-fruiting adult trees with an average height ranging from 5 to 8 m. Over half of the trees (22 individuals) exhibited dry branches and foliar damage caused by insect activity and pathogenic infection. Moderate infestations were by *M. mumecola*, and incidences of *Hyphantria cunea* (fall webworm). Other plant species observed at the collection site included *Berberis heteropoda*, *Cotoneaster melanocarpus* Fisch. ex Blytt, *Lonicera xylosteum* L., and *Picea schrenkiana* subsp. *tianschanica*.

Site 2, located in Bolshoy Kyrgyzsay Gorge (Uyghur District), comprised 24 accessions of the second *P. armeniaca* population. The population consisted primarily of diseased adult trees (4–10 m tall), exhibiting visible decline and no fruiting. All accessions were infested with aphids (*M. mumecola*), lichens, and pests including spider mites (*Tetranychidae* spp.), codling moth (*C. pomonella*), and fall webworm (*H. cunea*). Young trees were scarce, suggesting limited natural regeneration, with the few present already affected by pests and diseases. The surrounding plant species at the collection site included *Berberis heteropoda*, *Malus sieversii* (Ledeb.) M.Roem., *Lonicera xylosteum*, *Picea schrenkiana* subsp. *tianschanica*, *Filipendula ulmaria* (L.) Maxim., and *Cotoneaster melanocarpus*.

Site 3, located in the foothills of Bolshoy Kyrgyzsay Gorge (Uyghur District), comprised 23 accessions of the second *P. armeniaca* population, primarily fruit-bearing adult and young trees. These ranged from medium to tall stature, with heights averaging 4–8 m. Disease incidence was low, primarily due to rust diseases (*Puccinia* spp.). Pest infestations were minimal, with aphids (*M. mumecola*) predominant. Both adult and young trees exhibited abundant fruiting, producing oval, yellow drupes. Surrounding plant species included *Berberis heteropoda* Schrenk, *Malus sieversii* (Ledeb.) M.Roem., and *Ulmus pumila* L.

Site 4, located in Chundzha Village within Karadala Forestry (Uyghur District), comprised five accessions of the second *P. armeniaca* population, including three adult trees (~5 m tall) and two young trees (~3 m tall). No pests or diseases were observed, and all trees appeared healthy with active fruiting. Dominant surrounding vegetation included *Artemisia santolinifolia* (Turcz. ex Pamp.) Krasch., *Glycyrrhiza uralensis* Fisch., *Malus sieversii* (Ledeb.) M.Roem., *Melica transsilvanica* Schur, and *Rosa platyacantha* Schrenk.

The expeditions across the Almaty region identified numerous *P. armeniaca* individuals but underscored the vulnerability of this genetic resource to multiple threats, including diseases, pests, and natural aging of trees. Additionally, recurring climatic events, such as late frosts during flowering and extreme temperatures prevalent in the region, have accelerated population decline. Consequently, the genetic diversity of *P. armeniaca* faces significant risk of loss in the wild, necessitating urgent conservation efforts. Developing complementary ex situ conservation strategies is essential to safeguard this genetic diversity and ensure its long-term preservation and availability for future use [21,22,23,24,25,26,34,91].

### 2.2. Remote Sensing of Territories for Mapping Natural Populations of P. armeniaca

**Figure 1:**
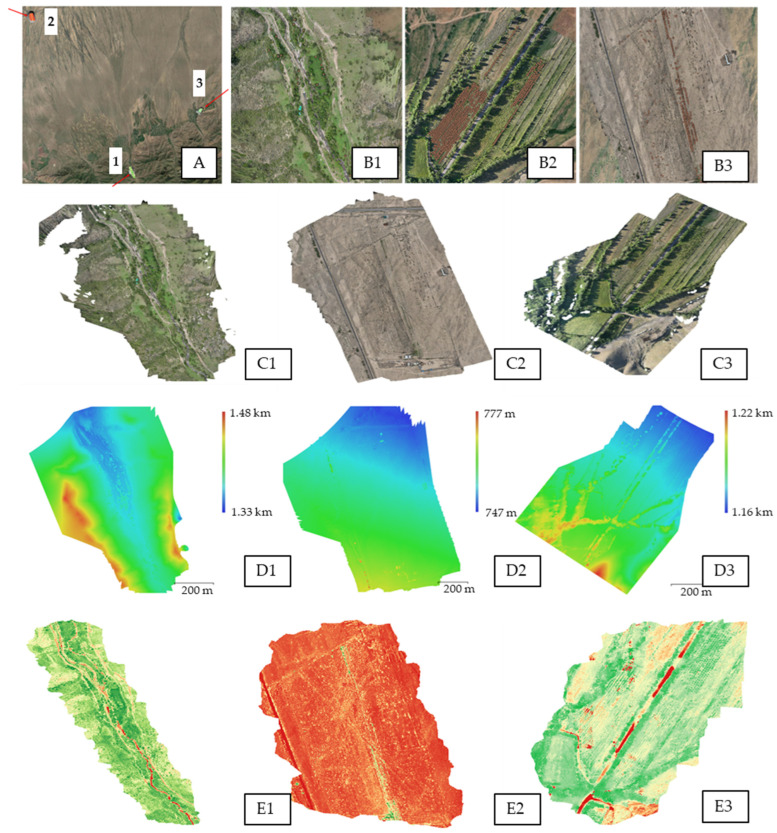
Remote sensing analysis of *Prunus armeniaca* growing areas in Bolshoe Aksu Gorge, Uyghur District, Almaty region. (**A**) Satellite map showing digitized plots 1, 2, and 3 marked by red arrows; scale 1:10,481 (estimated), height 120 m. (**B1**–**B3**) Growth maps with *Prunus armeniaca* marked by red dots corresponding to plots in (**A**), captured at 80–120 m altitude. (**C1**–**C3**) Orthophotomaps of the plots. (**D1**–**D3**) Digital Elevation Models (DEMs) with elevation ranges (e.g., 1.33 m to 1.77 m). (**E1**–**E3**) Normalized Difference Vegetation Index (NDVI) maps indicating vegetation quality and quantity.

Expeditions in 2023 revealed a significant concentration of wild *P. armeniaca* in Bolshoy Aksu Gorge, Uyghur District, designated as Site 1 of the second population. Based on this observation, three study areas were established within the gorge for remote sensing analysis (Figure 1A), with dimensions: (1) 750 × 850 m (Figure 1(B1)); (2) 850 × 900 m (Figure 1(B2)); and (3) 830 × 700 m (Figure 1(B3)). In 2024, satellite imagery of these areas was acquired and processed to generate detailed map sets of the surveyed regions.

Orthophotoplans were generated for the three study plots in Bolshoy Aksu Gorge, as shown in Figure 1(C1–C3). These high-resolution, georeferenced photographic representations, derived from processed overlapping aerial imagery [93], enabled precise delineation of *P. armeniaca* distribution and supported the creation of geodetic materials, including topographic and site maps. Additionally, they facilitated detailed assessments of vegetation structure across the plots. Analysis revealed that *P. armeniaca* trees form localized clusters of 5–10 individuals within ~50 m^2^ areas, with inter-cluster distances ranging from 20 to 50 m, indicating a mosaic distribution pattern. Visual analysis of orthophotoplans showed vibrant green foliage in plots 1 and 3, suggesting healthy plant physiology, whereas plot 2 exhibited paler and darker foliage, potentially indicating stress, disease, or moisture deficiency. Orthophotoplans are valuable for applications such as land-use management, ecological research, and vegetation health monitoring, including detecting plant disease outbreaks [93,94,95,96,97,98].

To enhance habitat analysis, orthophotoplans were integrated with a Digital Elevation Model (DEM) for the three study plots in Bolshoy Aksu Gorge (Site 1, second *Prunus armeniaca* population). The DEM, constructed from elevation point arrays excluding vegetation and anthropogenic structures (Figure 1(D1–D3)) [93,99], represented the plots as three-dimensional terrain models. Plot 1 ranged from 1330 to 1480 m above sea level, plot 2 from 747 to 777 m, and plot 3 from 1160 to 1220 m. Combining the DEM with orthophotoplans enabled detailed assessment of topographic features, including slope, elevation, and relief, influencing soil moisture, heat, and nutrient distribution. Plot 2, located at a significantly lower elevation with minimal slope compared to plots 1 and 3, likely experienced microrelief conditions contributing to the observed physiological stress in its vegetation. The DEM serves as a foundation for digital relief maps in geographic information systems (GIS) and supports landscape modeling, including applications in land-use studies, geological analyses, flood, and drainage modeling [40,41,45,99].

Vegetation indices, widely used remote sensing tools, enable analysis of vegetation dynamics [100]. These indices monitor plant health, estimate green biomass, and assess land-use and land-cover changes over time [101,102,103]. Various indices target specific vegetation characteristics, derived from spectral data across different wavelength ranges [103,104,105,106]. The Normalized Difference Vegetation Index (NDVI), a commonly applied index, characterizes canopy growth, vigor, and overall plant health [107,108]. NDVI distinguishes vegetated from non-vegetated areas, identifies vegetation types, and assesses health status [109]. Calculated from satellite-derived reflectance in the red and near-infrared (NIR) bands, NDVI leverages the fact that healthy vegetation absorbs red light due to chlorophyll and reflects NIR radiation. In contrast, unhealthy or sparse vegetation reflects more red light and less NIR radiation. NDVI values range from −1 to 1, with values above 0.6 indicating moderate to dense healthy vegetation [110,111,112].

The NDVI values obtained from the three study plots indicated that flight altitude (80 m vs. 100 m above ground level) had no significant effect on the results (Table 1). Minimal differences in NDVI values were observed between images captured at the two altitudes. These results suggest that moderate variations in flight altitude do not compromise the reliability of the NDVI under the tested conditions. Plot 2 recorded the lowest NDVI values (0.33–0.34), consistent with field observations of vegetation stress and visible damage. These values reflect sparse or weakened plant cover with reduced photosynthetic activity. In contrast, plots 1 and 3 exhibited higher NDVI values (0.61–0.63 and 0.68–0.70, respectively), indicative of moderate to dense healthy vegetation [110,111,112].

NDVI data provide valuable insights to support decision-making in conservation and management strategies. One of its key applications is the monitoring of vegetation status over time [102,113]. NDVI facilitates evaluation of vegetation responses to environmental stressors across spatial scales, from regional to global [49,111,114,115]. Integrating UAVs equipped with multispectral sensors has further enhanced both the spatial and temporal resolution of NDVI data, supporting rapid, non-destructive, and cost-effective vegetation assessments at the landscape level [116,117]. In this study, NDVI values derived from UAV-acquired multispectral imagery at two flight altitudes (80 m and 100 m) provided high-resolution, site-specific data that revealed differences in vegetation condition among the study areas in the Bolshoy Aksu Gorge. This approach offers an effective tool for identifying conservation priorities and guiding land management interventions.

### 2.3. Population Genetic Diversity of P. armeniaca Using SSR Markers

DNA barcoding using the chloroplast regions *matK*, *rbcL*, and *trnH-psbA* was conducted for all 54 accessions. Sequence analysis confirmed the identity of all samples as *Prunus armeniaca* through comparison with reference sequences available in GenBank. Following taxonomic verification, phenotypic evaluation of *P. armeniaca* accessions from the Turgen Gorge, Bolshoy Aksu Gorge, and Bolshoy Kyrgyzsay Gorge revealed the presence of two distinct populations. A total of 54 accessions, initially assigned to these two populations based on morphological traits, were subsequently analyzed using molecular markers. Genotyping with 13 SSR markers revealed a high level of genetic diversity among the accessions, which was not reflected in the initial morphological classification.

Clustering based on 13 SSR markers identified 11 genetically distinct populations, contrasting with the two populations initially classified by morphological traits. Similar discrepancies have also been reported by other authors and are often attributed to environmental influences, phenotypic plasticity, and the limitations of morphological descriptors in capturing genetic variation [118,119,120,121,122,123,124,125,126]. These findings underscore the limitations of relying solely on morphology for species identification as well as population classification. They also highlight the critical role of molecular tools in accurate genetic assessment for effective conservation, breeding, and germplasm management [54,127,128,129]. Moreover, this genetic structuring also raises important considerations regarding how we define and prioritize populations, subpopulations, and accessions in the context of conservation. While ex situ conservation programs typically focus on preserving individual accessions or clonal lines, our study addresses naturally occurring populations, which may consist of multiple, genetically and spatially distinct subpopulations within the same spatial area. The selected samples represent natural populations from three regions (Turgen, Bolshoy Aksu, and Bolshoy Kyrgyzsay Gorges) with distances between them ranging from 10 to 180 km. Within these regions, wild apricot trees tend to occur in localized patches. Although our study did not formally quantify the number of individuals per patch or the spatial extent of each group, field observations and aerial imagery suggest that apricot trees often form small to medium-sized clusters. Incorporating molecular data into conservation planning enables more targeted and efficient strategies that capture the full range of genetic diversity present in wild populations.

Genetic diversity analysis of 54 individuals across 11 populations revealed substantial variation in genetic parameters (Table 2). Sample sizes per population (N) ranged from 1 to 18, with an average of 4.91. The number of alleles (Na) ranged from 1.692 in Pop6 (Bolshoy Kyrgyzsay) to 5.846 in Pop2 (Bolshoy Aksu), while the effective number of alleles (Ne) varied between 1.692 and 3.671, with Pop4 (Turgen Gorge) showing the highest value. These findings, along with the observed discrepancy between morphological and molecular clustering, underscore the conceptual differences among accessions, subpopulations, and populations. In the context of conservation, a population is typically defined as a group of interbreeding individuals forming a reproductive unit. Subpopulations may represent semi-isolated genetic clusters within a broader population, while accessions often correspond to individual plants or clonal lines preserved ex situ [32,38].

The values of the Shannon index (I), which reflects the level of genetic diversity, ranged from 0.480 (Pop6) to 1.398 (Pop4), indicating variable genetic richness among populations. Observed heterozygosity (Ho) ranged from 0.615 to 0.904, with the highest value recorded in Pop10 (Bolshoy Aksu). Expected heterozygosity (He) varied from 0.346 and 0.707, and unbiased expected heterozygosity (uHe) ranged from 0.616 to 0.800, reflecting differences in allelic evenness and sample sizes.

The inbreeding coefficient (F) exhibited both positive and negative values across populations, ranging from 1.000 in Pop3 and Pop6 to −0.447 in Pop9 (Table 2). Positive F values indicate a heterozygote deficit or potential inbreeding, while negative values suggest heterozygote excess [118,130]. Most populations showed negative F values, with the lowest in Pop9 (−0.447), Pop10 (−0.341), and Pop7 (−0.338), suggesting relatively high genetic diversity and low inbreeding in those populations.

Populations from Bolshoy Aksu Gorge exhibited the highest overall allelic diversity among the gorges. Pop4 (Bolshoy Aksu Gorge) showed the highest effective number of alleles (Ne, 3.671) and Shannon index (I, 1.398) and the second-highest number of alleles (Na, slightly below Pop2, also from Bolshoy Aksu Gorge). These results indicate a genetically rich and diverse population. In contrast, Pop6 (Bolshoy Kyrgyzsay Gorge) had the lowest diversity indices (Na: 1.692, I: 0.480), suggesting reduced genetic variability, likely due to small population size or isolation (Table 2).

Average genetic diversity parameters across 13 microsatellite loci and populations show a moderately high level of genetic variation in the studied *P. armeniaca* populations (Table 2). The overall average number of observed alleles (Na) was 3.776, and the effective number of alleles (Ne) was 2.866. This relatively high NE value suggests a sufficient level of uniformity of allele distribution and the absence of allelic dominance within populations. The Shannon index (I), averaged 1.087, also confirms the presence of diversity at the allelic composition level. Observed heterozygosity (Ho, 0.759) exceeded expected heterozygosity (He, 0.596). This difference is confirmed by a negative inbreeding coefficient (F = −0.303), which indicates a higher-than-expected level of heterozygosity. This result may be attributed to cross-pollination, which promotes genetic recombination and maintains heterozygosity [131]. In addition, a high degree of gene introgression between wild and cultivated *P. armeniaca* in Central Asia has been documented, contributing to increased genetic diversity in the species [11,132]. Furthermore, migration between genetically distinct populations can alter population structure and promote heterozygosity [133].

These mechanisms are consistent with the diversity levels observed in the present study and align with previous findings on wild *Prunus armeniaca* populations in Central Asia. For example, Hu et al. [134] reported He = 0.610 and I = 1.22 in wild apricots from the Tien Shan Mountains, confirming a similarly high diversity level. In contrast, Sheikh et al. [4] observed significantly lower values (He ≈ 0.45) in cultivated Himalayan accessions. A comparable pattern was found by Bourguiba et al. [135], who reported a geographical gradient in Mediterranean apricots, with expected heterozygosity decreasing from the east (He = 0.653 in Cluster 1) to the southwest (He = 0.508 in Cluster 4). These comparisons emphasize the importance of conserving local wild apricot populations as reservoirs of genetic variation for breeding and long-term sustainability.

Overall, the balanced genetic structure and high heterozygosity observed suggest that these populations have retained evolutionarily significant diversity, which is crucial for long-term resilience and adaptability. This level of genetic variation highlights the potential of these populations to serve as robust genetic resources for crop improvement, restoration, and adaptation to changing environmental conditions. Therefore, integrated in situ and ex situ conservation approaches are crucial to safeguard rare and potentially adaptive allelic variants that may be essential for meeting future needs [34,54,136,137,138,139,140].

Standard F-statistics (Fis, Fit, Fst) and the estimated number of migrants per generation (Nm) were calculated for each of 13 microsatellite loci to assess population structure and genetic exchange among *P. armeniaca* populations (Table 3). Analysis of molecular variance (AMOVA) revealed moderate genetic differentiation, with a mean Fst of 0.220 ± 0.015, indicating that 22% of genetic variation occurs among populations and 78% is found within them. This level of differentiation, consistent with cross-pollinated species maintaining moderate gene flow [141], reflects a moderate population structure. Nm ranged from 0.537 to 1.745 (mean: 0.961 ± 0.094), suggesting limited gene exchange (Nm < 1) between populations. A critical threshold of Nm > 1 is generally required to prevent population differentiation due to genetic drift [142]. Therefore, for most loci, migration between populations is insufficient to completely equalize their gene pools. However, higher Nm values at certain loci (Locus5: Nm = 1.745; Locus3: Nm = 1.435) indicate higher levels of gene migration and therefore, this suggests that occasional gene flow does occur.

Consistently negative Fis values across all loci (mean of −0.274) indicate heterozygote excess within populations (Table 3). This may reflect frequent cross-pollination, heterosis, or mating between genetically distinct individuals, possibly following secondary contact between previously isolated populations. Fit values, representing overall heterozygosity across populations, ranged from −0.194 to 0.191, with an overall mean of 0.005 ± 0.032. These values indicate that observed heterozygosity aligns with random mating expectations despite local genetic structure. In summary, combined analysis of F-statistics and the Nm parameter confirms a moderately structured population system with limited gene flow. The observed excess heterozygosity within populations is potentially driven by mixed or unstable reproductive systems [143,144].

**Figure 2:**
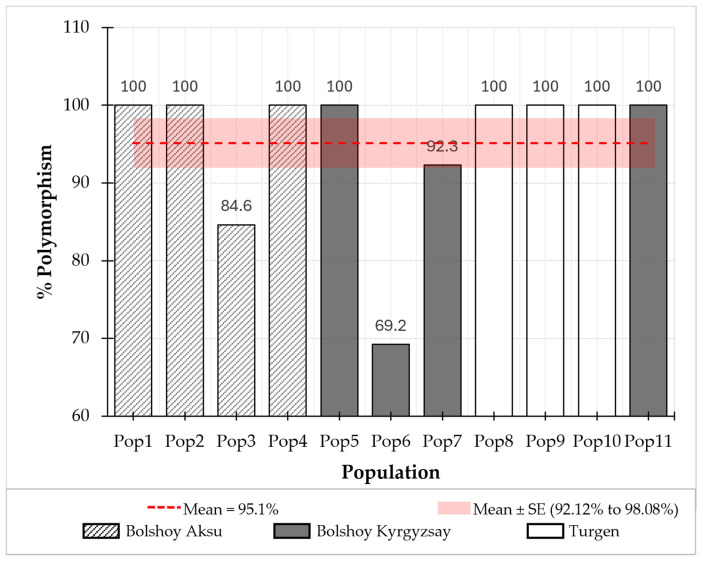
Percentage of polymorphic loci across 11 *Prunus armeniaca* populations from three locations in the Almaty region, Kazakhstan.

The percentage of polymorphic loci was assessed across 13 microsatellite loci for 54 *Prunus armeniaca* individuals from 11 populations sampled from three distinct locations in the Almaty region. The analysis showed a high level of genetic variability among all populations (Figure 2). Eight populations (1, 2, 4, 5, 8, 9, 10, 11) showed 100% polymorphism, indicating at least two alleles per locus and high genetic diversity. Populations 3 and 7 had polymorphism levels of 84.62% and 92.31%, respectively, while Pop6 had the lowest (69.23%), still reflecting considerable variation. The reduced polymorphism Pop6, when compared to other populations, may be attributed to a limited population size [145,146] or geographic isolation restricting gene flow [147,148,149]. Isolated and fragmented populations are generally more vulnerable to both biotic and abiotic stresses, which makes them more prone to genetic erosion [150,151]. The observed decline in genetic diversity within such populations underscores the urgent need for ex situ conservation strategies to prevent the loss of these genetic resources.

The high level of polymorphism observed in *P. armeniaca* populations from the Almaty region indicates a significant genetic resource reservoir that is valuable for both conservation and breeding efforts [11,118,152]. Furthermore, these findings confirm the effectiveness of the selected microsatellite markers in assessing intrapopulation diversity and interpopulation differentiation.

### 2.4. Population Genetic Structure of P. armeniaca

**Figure 3:**
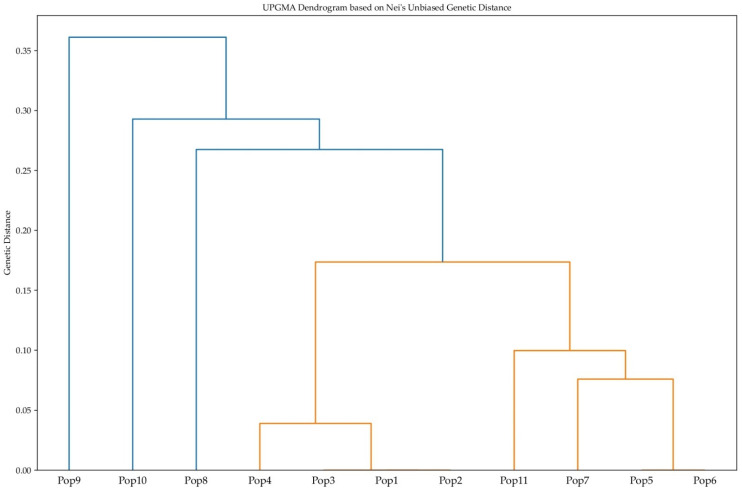
UPGMA dendrogram constructed based on Nei’s Unbiased Genetic Distance, illustrating the genetic relationships among 11 populations of *Prunus armeniaca*. Cluster I includes Pop8, Pop9, and Pop10 (Turgen Gorge); Cluster II comprises Pop1, Pop2, Pop3, and Pop4 (Bolshoy Aksu Gorge); and Cluster III contains Pop5, Pop6, Pop7, and Pop11 (Bolshoy Kyrgyzsay Gorge).

The phylogenetic tree was constructed using the UPGMA (Unweighted Pair Group Method with Arithmetic Mean) algorithm, based on Nei’s Unbiased Genetic Distance matrix calculated from microsatellite data (Figure 3).

The UPGMA tree showed that 11 populations of *P. armeniaca* can be clearly divided into three distinct genetic clusters, which correspond to their geographic origins. The first cluster includes Pop8, Pop9, and Pop10 from the Turgen Gorge; the second cluster comprises Pop1, Pop2, Pop3, and Pop4 from the Bolshoy Aksu Gorge; and the third cluster contains Pop5, Pop6, Pop7, and Pop11 from the Bolshoy Kyrgyzsay Gorge (Figure 3). These findings suggest frequent gene flow within gorges but limited gene flow across them, consistent with moderate genetic differentiation and low migration (Table 3). Pop9 is positioned as the most genetically distant population in the dendrogram, possibly due to geographic remoteness or ecological isolation within Turgen Gorge. Despite its distinctiveness, Pop9 retains a degree of biological connectivity with other Turgen Gorge populations. Its divergent position highlights its potential as a reservoir of unique genetic variation for conservation efforts.

The grouping of Pop4, Pop3, Pop1, and Pop2 into a linear phylogenetic arrangement suggests a gradual accumulation of differences or a series of migration/dispersal events.

Pop 5 and Pop 6 are the most closely related, indicating their recent common origin or intensive gene exchange. Pop 7 joins this pair, forming a subcluster, which may indicate the geographical proximity of these three populations. In addition, the pairs Pop1–Pop2 and Pop11–Pop7 show relatively low genetic distances, which also indicates a high degree of genetic relationship. Although some populations, such as Pop1 and Pop2, exhibited extremely short genetic distances, the grouping into 11 populations was retained to reflect the original sampling design based on distinct geographic locations within the gorges. Each population represents individuals collected from separate micro-sites, and while some groups are genetically very similar, spatial separation and potential microecological differences justify their treatment as separate population units. This approach enables a more detailed analysis of genetic structure and is especially relevant for conservation strategies, where the preservation of spatially structured diversity may be as important as the preservation of intrinsic genetic diversity.

Clustering of the 11 populations using UPGMA based on Nei’s Unbiased Genetic Distance further confirmed the observed genetic structure. Some populations (e.g., Pop2, Pop3, and Pop5) exhibited extremely short branch lengths in the dendrogram, reflecting minimal, but not zero, genetic distances to neighboring clusters. These short distances indicate high genetic similarity. Although such branches may visually appear disconnected due to scaling, they are part of a continuous hierarchical tree structure.

Overall, these findings have significant implications for the management and conservation of *P. armeniaca* genetic resources within Almaty region. The observed genetic structure highlights the importance of implementing site-specific conservation strategies, particularly for genetically distinct and isolated populations that may harbor unique allelic variants of potential significance for crop improvement. Thus, this study provides a valuable foundation for identifying and prioritizing conservation targets, confirming genetic redundancies, and verifying uniqueness, thereby enhancing the efficiency and effectiveness of future conservation efforts for *P. armeniaca* in the region. DNA barcoding provided reliable confirmation of species identity for all accessions. Although chloroplast markers were not used to assess intraspecific genetic variation in this study, their use was valuable to validate the morphological classification and ensure taxonomic accuracy prior to microsatellite analysis.

Furthermore, the distinction between accession, subpopulation, and population has direct implications for conservation strategies. Our results demonstrate that genetically distinct units identified by SSR markers may correspond to subpopulations or local populations with unique evolutionary significance. The identified genetic groups varied in size, ranging from single individuals to larger clusters within natural habitats. These findings emphasize the need to integrate molecular data into conservation planning to ensure that both broad population structures and fine-scale genetic diversity are adequately represented.

## 3. Materials and Methods

### 3.1. Plant Material and Botanical Description

**Figure 4:**
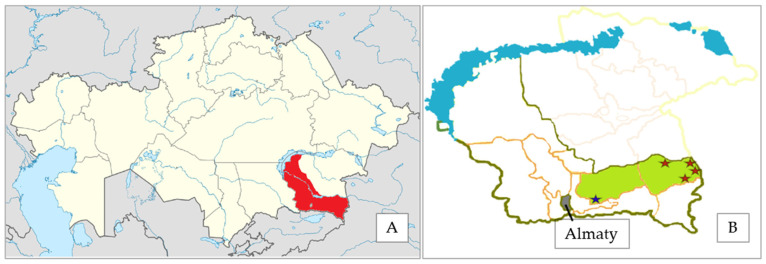
Collection sites of *Prunus armeniaca* accessions during field expeditions in Kazakhstan, 2023–2024. (**A**) The Almaty region, where collection sites are located, is highlighted in red on the map of Kazakhstan [155]. (**B**) Collection sites within the region are marked with colored stars: a blue star indicates Turgen Gorge (Enbekshikazakh District), and red stars denote the four sites in Uyghur District—Bolshoy Aksu Gorge (Site 1), Bolshoy Kyrgyzsay Gorge (Site 2), Bolshoy Kyrgyzsay Gorge foothills (Site 3), and Chundzha Village (Site 4).

Plant material from 111 *Prunus armeniaca* individuals was collected from two populations in the Almaty region, Kazakhstan, during expeditions in 2023–2024. Botanists identified individuals using 46 standardized morphological descriptors (Section 3.2), assigning them to populations based on geographic origin. Populations were separated by at least 180 km, with individuals within a population sampled at least 2 m apart. A population refers to groups of individuals initially classified based on field observations and morphological descriptors. Population 1 included 23 individuals from Turgen Gorge, Enbekshikazakh District. Population 2 comprised 88 individuals from four sites, 10–40 km apart, in Uyghur District and Karadala Forests: 43 individuals from Bolshoy Aksu Gorge (Site 1), 24 from Bolshoy Kyrgyzsay Gorge (Site 2), 16 from Bolshoy Kyrgyzsay Gorge foothills (Site 3), and 5 from Chundzha Village (Site 4). A collection site refers to a specific geographic location where samples were collected. Figure 4 shows the collection sites where the plant material was collected. An interdisciplinary team of taxonomists and geobotanists oversaw species identification. Plants grew on well-drained mountain brown soils [153,154]. The average monthly air temperature at the collection sites ranges from 8 to 40 °C in the summer (June to August) and from −2 to −22 °C in the winter (December to March), with an average annual precipitation of 850–900 mm.

The selection of plant material was not intentional, but situational, i.e., plant material was collected from trees at the collection sites based on actual field encounters [34]. Geographical coordinates were recorded using the eTREX^®^H Garmin Montana 750i GPS navigator (Garmin Ltd., Olathe, KS, USA) along with observations of each accession collected in situ. Each accession was labeled with a unique tag containing the collection number and date. The sizes (height or width) of the tree accessions were determined using a standard tape measure. The presence of diseases and pests was assessed visually. In addition, photographs were taken using a Canon EOS RP RF 24-105 F4-7.1 IS STM camera (Canon Inc., Tokyo, Japan) and an iPhone 14 Pro mobile phone for further evaluation. Within a 10 m radius of each accession, 5 to 10 species of surrounding plants were recorded. Table 4 lists the collection sites, GPS coordinates, and elevation of the exact collection point.

Collections of plant material were made of leaves and shoots. Random samples of leaf blades (10 pieces) were collected from each accession in July–August. Leaves were placed between two layers of filter paper, placed in hermetically sealed bags containing 20 g of silica gel, and transported to the laboratory. Leaves were stored at room temperature and used for species characterization and DNA extraction. Simultaneously with the leaf collection, a few shoots (30 cm) were collected from each accession and placed between thick, folded sheets of cardboard (42 × 59.4 cm) and used for herbarium preparation. Additionally, depending on availability, a sample of mature fruit was collected during the fruiting season, which ranged from July to August. Fruits were carefully placed in 250 mL plastic or polyethylene containers, transported to the laboratory, stored at room temperature, and used within 5 days of collection.

### 3.2. Botanical Description

The population status of *Prunus armeniaca* was assessed using descriptors established by leading international organizations, including the Food and Agriculture Organization of the United Nations [31], the International Plant Genetic Resources Institute (IPGRI), the European Cooperative Programme for Plant Genetic Resources, and research by Lateur et al. [32] and Rotach [33]. The evaluation encompassed 46 parameters, as detailed in Supplementary Table S1. This field-based classification served as the basis for initial population assignments prior to molecular analysis.

### 3.3. Herbarium Production

**Figure 5:**
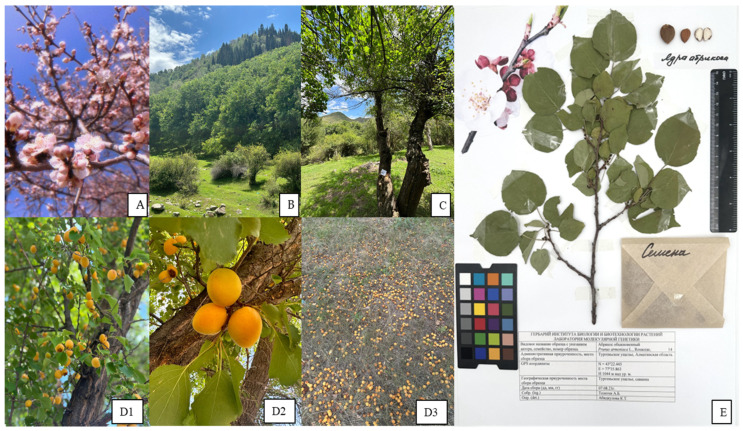
Natural populations of *Prunus armeniaca* conserved in in situ conservation sites in Kazakhstan, Almaty region. (**A**): Plants flowering in the Turgen Gorge, Enbekshikazakh district; (**B**): Plants in the Bolshoy Aksu Gorge, Uyghur district; (**C**): A viable, non-fruit-bearing adult, and tree in the Bolshoy Kyrgyzsay Gorge, Uyghur district; (**D1**,**D2**): A viable, fruit-bearing adult tree and fallen fruit on the ground (**D3**) in the foothills of the Bolshoy Kyrgyzsay Gorge, Uyghur district; (**E**): Representation of a herbarium collection for a *P. armeniaca* accession.

Herbaria of *P. armeniaca* (Figure 5) were prepared using shoots, fruits cleaned of pulp, seeds, and a photograph of the specimen during flowering. The herbarium description included the species name, specimen number, collection date, location, GPS coordinates, and the collecting researcher’s name. Herbarium photographs documented dimensions with a ruler (in centimeters) and color using the Datacolor SCK300 Spyder Checkr Photo Color Chart (Datacolor Inc., Lawrenceville, NJ, USA). Photographs were taken in a Godox LSD60 photo box with LED lighting (Godox Photo Equipment Co., Ltd., Shenzhen, China) using a Canon EOS RP RF 24-105 F4-7.1 IS STM camera (Canon Inc., Tokyo, Japan). Images with 3× magnification were captured using a Levenhuk ZOOM 0750 stereoscopic microscope and a Levenhuk M200 digital camera (Levenhuk Inc., Tampa, FL, USA).

### 3.4. Remote Sensing of Territories for Mapping Natural Populations of P. armeniaca

The field survey was conducted in July 2024 across three sections of Bolshoe Aksu Gorge, Uyghur District (Population 2, Site 1). The study area was delineated along the contours of the territory, and unmanned aerial vehicles (UAVs) were prepared for photography. Aerial photographs were captured during daytime under favorable weather conditions at altitudes of 80–120 m. Remote sensing utilized the following UAVs: (1) DJI Mavic 3E with an RGB camera, and (2) DJI Phantom 4 with a multispectral camera, capable of 20 MP JPEG photos and 5 MP TIFF images, respectively [156]. The maximum image resolutions were 5280 × 3956 pixels for the RGB channel and 2592 × 1944 pixels for the multispectral channel, with an equivalent focal length of 24 mm. Monochrome sensors captured images in spectral ranges: green (G 560 ± 16 nm), red (R 650 ± 16 nm), red edge (RE 730 ± 16 nm), and near-infrared (NIR 860 ± 26 nm) channels [157]. Relief maps were generated in GeoTIFF format, 3D terrain models in OBJ format, and a project in QGIS, marking wild *P. armeniaca* locations [94,158,159,160,161,162]. The aircraft route was designed to ensure 60% vertical and horizontal image overlap [40,41,99]. Original UAV camera data were provided as photographs. Open-source satellite data were utilized for analysis, including the Esri World Imagery base map, which offers satellite and aerial imagery at resolutions of 1 m or better, sourced from Sentinel, Landsat, and MODIS satellites. The map scale was 1:104,819, with a height of 20 km.

The Normalized Difference Vegetation Index (NDVI), a numerical indicator of vegetation quality and quantity over a surface area, was calculated using the formula: (NIR − RED)/(NIR + RED), where NIR represents near-infrared reflectance and RED denotes red reflectance [49,50,111,163,164].

### 3.5. Molecular Analysis

A total of 54 *P. armeniaca* accessions, initially classified into two populations based on morphological descriptors, were analyzed using microsatellite markers. These accessions were randomly selected from a broader set of 111 individuals previously evaluated for morphological traits. The selected samples represent natural populations from three geographic regions: Turgen Gorge (13 accessions), Bolshoy Aksu Gorge (Site 1, 28 accessions), and Bolshoy Kyrgyzsay Gorge (Site 2, 13 accessions). The GPS coordinates and elevations from which the 54 *P. armeniaca* accessions were collected for molecular analysis are presented in Supplementary Table S2. The term genetic cluster is used later in the results to describe groupings identified through SSR-based analysis.

#### 3.5.1. DNA Extraction

The collected leaves were placed in a paper envelope and dried in a dry-heat oven at 50 °C for 3 h. A 0.1 g sample of dried leaf was placed in a 2 mL tube containing two metal balls of 4 mm diameter and ground in a Tissue Lyser II homogenizer (Qiagen, Hilden, Germany) for 10 min. DNA was extracted from the ground leaf tissue using the GeneJET Plant Genomic DNA Purification Mini Kit (ThermoScientific, Waltham, MA, USA). DNA extraction was performed according to the manufacturer’s instructions. DNA concentration was measured using a Qubit 2.0 fluorimeter (Life Technologies, Tecan, Grödig, Austria) using the Qubit dsDNA BR Assay Kit (Invitrogen™, Carlsbad, CA, USA).

#### 3.5.2. PCR Setup

For biodiversity studies of rare and endangered *P. armeniaca* (apricot), DNA barcoding using the chloroplast genes (*matK*, *rbcL*, *trnH-psbA*), along with genomic marker internal transcribed spacer (*ITS*), was performed. PCR was performed using primers listed in Table 5. PCR reaction with total volume of 30 µL contained 1x PCR Gold Buffer, 0.2 mM of each dNTP, 2.5 mM MgCl_2_, forward and reverse primers to final concentration of 0.4 µM, 1 unit per reaction of AmpliTaq Gold DNA polymerase (Applied Biosystems, Waltham, MA, USA), 5 µL of isolated DNA and water to reach a final volume of 30 µL. The PCR reaction was performed on a SimpliAmp™ Thermal Cycler GeneAmp PCR amplifier (Applied Biosystems, Singapore). The PCR program included preliminary denaturation at 95 °C for 10 min; 33 cycles with denaturation at 95 °C for 15 s, primer annealing for 40 s at the temperature given in Table 5, and elongation at 72 °C for 40 s; and final elongation at 72 °C for 10 min.

#### 3.5.3. Electrophoretic Accounting of PCR Amplification

PCR products were separated in 1.5% agarose gel prepared in 1× TAE buffer (40 mM Tris, 20 mM acetate, and 1 mM EDTA at pH 8.6) with intercalating dye—ethidium bromide. Electrophoresis was carried out in a PowerPac horizontal electrophoresis chamber using a BioRad Electrophoretic bath current source (Bio-Rad Laboratories, Inc., Hercules, CA, USA). 1× TAE buffer was used as an electrode buffer. The results obtained were visualized using the Gel Doc gel documentation system (Bio-Rad) with QuantityOne software (Bio-Rad) and analyzed with Quantity One software version 4.6.9 (Bio-Rad). The size of the PCR product was determined relative to the molecular weight of the DNA marker Step 100 Long (BiolabmixLLC, Moscow, Russia).

#### 3.5.4. Purification of PCR Products and Sequencing

PCR products were purified using magnetic beads as previously described [169]. Sequencing reactions were performed using the BigDye^®^ Terminator v3.1 Cycle Sequencing Kit (Thermo Fisher Scientific, Vilnius, Lithuania) according to the manufacturer’s instructions, followed by fragment separation on an Applied Biosystems^®^ 3500 automated genetic analyzer (Applied Biosystems, Waltham, MA, USA). The nucleotide sequences obtained with the forward and reverse primers were analyzed and combined into a common sequence using the SeqMan software version 7.1.0 (Lasergene, DNASTAR) [170].

#### 3.5.5. SSR Labeling of the Biodiversity of Rare and Endangered *P. armeniaca*

PCR reaction was performed in a volume of 20 μL and included 10 μL Biomaster HS-Taq PCR (2×) (Biolabmix, Russia), 1 μL each of forward and reverse primers (Table 6) at a concentration of 5 pmol/μL, DNA 36 ng, and water up to 20 μL. PCR cycling program was performed on SimpliAmp™ Thermal Cycler (Applied Biosystems) and included the following steps: preliminary denaturation at 95 °C for 5 min; 32 cycles of denaturation at 95 °C for 25 s, primer annealing at 55 °C for 1 min, elongation at 72 °C for 1 min and 30 s, and a final elongation, including 1 step at 68 °C for 30 min. After PCR amplification, the PCR products were mixed in three reaction mixtures: the first mixture included 5 μL of PCR products obtained with primers Pr-1, Pr-10, Pr-13, Pr-3, Pr-4, Pr-5, Pr-6, and Pr-7; the second mixture contained 20 μL of water and 5 μL of PCR products obtained using primers Pr12, Pr15, and Pr2; the third reaction mixture contained 15 μL of water and 5 μL of PCR products obtained using primers Pr11, Pr5, Pr8, and Pr9. The resulting mixtures in a volume of 1.6 μL were transferred to 10 μL of formamide with a LIZ 500 size standard and separated by capillary electrophoresis. SSR profile analysis was performed using GeneMapper software version 4.0 (Applied Biosystems).

#### 3.5.6. Data Analysis

Microsatellite markers (SSR) were used for the analysis of genetic diversity, and data were processed using GenAlEx version 6.5 [175]. Based on the initial matrix of allelic data, the main population genetic parameters were calculated, including the number of alleles (Na), the effective number of alleles (Ne), the Shannon index (I), the observed (Ho) and expected (He) heterozygosity, the unbiased expected heterozygosity (uHe), the inbreeding coefficient (F), and the percentage of polymorphic loci (%P). Calculations were carried out for each locus and population separately, as well as averaged over all loci. To assess genetic differentiation between populations, Nei’s Unbiased Genetic Distance matrix of pairwise genetic distances was calculated and presented as a symmetric table. The matrix data were exported from GenAlEx and used to construct a cluster dendrogram using the unweighted pair group method with arithmetic mean (UPGMA) method. The UPGMA dendrogram was constructed in the Python environment using the NumPy version 1.24.2, SciPy version 1.10.1, and Matplotlib version 3.7.1 libraries [176]. In particular, the linkage function from scipy.cluster.hierarchy module was used for agglomerative clustering by average distance, and the dendrogram function was used to visualize the hierarchical structure. The input distance matrix was converted to condensed format using the squareform function from scipy.spatial.distance module.

## 4. Conclusions

Geobotanical and remote sensing analyses revealed a considerable number of *P. armeniaca* accessions across the Almaty region. Equally important, these analyses also highlighted the species’ vulnerability to pests, diseases, climatic events, and plant aging. Our findings demonstrate the necessity of integrated morphological and molecular analyses to effectively differentiate closely related accessions. Genetic analysis of 54 accessions using 13 SSR markers provided a clearer picture of the genetic structure and identified 11 genetically distinct populations characterized by high heterozygosity and substantial genetic variability. Eight of these populations exhibited 100% polymorphism, highlighting their potential as reservoirs of adaptive genetic diversity for breeding, restoration, and climate resilience. Cluster analysis revealed three major genetic groups aligned with geographic origin, suggesting limited gene flow across Gorges and greater connectivity within them. These findings underscore the need for site-specific conservation strategies, especially for genetically distinct, isolated populations with unique allelic profiles. This study provides a valuable foundation for prioritizing conservation targets, confirming genetic redundancies, and preserving genetic uniqueness to enhance the efficiency and effectiveness of the future conservation and use of *P. armeniaca* in the region. Future efforts should prioritize long-term conservation of this genetic diversity reservoir to support crop improvement.

## Figures and Tables

**Table 1:** Normalized Difference Vegetation Index (NDVI) values for three study areas based on unmanned aerial vehicles (UAV)-acquired multispectral imagery at two flight altitudes (80 m and 100 m).

Areas	Altitude (m)	Fight Duration	Number of Photos Taken	NDVI
1 *	80	19 min 23 s	325	0.61
	100	23 min 17 s	474	0.63
2	80	20 min 42 s	382	0.33
	100	26 min 05 s	512	0.34
3	80	24 min 30 s	404	0.7
	100	18 min 20 s	296	0.68

* The three sections across the Bolshoe Aksu Gorge in the Uyghur district of the Almaty region.

**Table 2:** Genetic diversity parameters for 54 *Prunus armeniaca* individuals representing 11 populations across the Turgen Gorge, Bolshoy Aksu Gorge, and Bolshoy Kyrgyzsay Gorge, based on genotyping with 13 microsatellite (SSR) loci.

Population	Location	N	Na	Ne	I	Ho	He	uHe	F
Pop1	Bolshoy Aksu, Site 1	3	3.769	3.226	1.214	0.615	0.667	0.800	0.104
Pop2	Bolshoy Aksu, Site 1	18	5.846	2.714	1.226	0.628	0.599	0.616	−0.054
Pop3	Bolshoy Aksu, Site 1	1	1.846	1.846	0.587	0.846	0.423	0.846	1.000
Pop4	Bolshoy Aksu, Site 1	6	4.923	3.671	1.398	0.744	0.707	0.772	−0.052
Pop5	Bolshoy Kyrgyzsay, Site 2	5	4.231	3.189	1.234	0.769	0.646	0.718	−0.191
Pop6	Bolshoy Kyrgyzsay, Site 2	1	1.692	1.692	0.480	0.692	0.346	0.692	1.000
Pop7	Bolshoy Kyrgyzsay, Site 2	3	3.385	2.892	1.044	0.769	0.577	0.692	−0.338
Pop8	Turgen	6	4.231	2.998	1.215	0.782	0.650	0.709	−0.206
Pop9	Turgen	3	3.308	2.678	1.052	0.872	0.603	0.723	−0.447
Pop10	Turgen	4	4.231	3.424	1.285	0.904	0.680	0.777	−0.341
Pop11	Bolshoy Kyrgyzsay, Site 2	4	4.077	3.198	1.223	0.731	0.656	0.750	−0.131
***Mean***		4.91	3.776	2.866	1.087	0.759	0.596	0.736	−0.303

He, expected heterozygosity (= 1 − ∑ pi2); Ho, observed heterozygosity = (number of heterozygotes/N); I, Shannon’s information index = −1 × Sum (pi × Ln (pi)); N, sample sizer; Na, number of different alleles; Ne, number of effective alleles (=1/(∑ pi2)); pop, population; uHe, unbiased expected heterozygosity = (2N/(2N − 1)) × He; F, fixation index = (He − Ho)/He = 1 − (Ho/He).

**Table 3:** F-Statistics and estimates of gene flow (Nm) for each SSR locus across all populations.

Locus	Fis	Fit	Fst	Nm
Locus1	−0.185	0.191	0.317	0.537
Locus2	−0.387	−0.079	0.222	0.876
Locus3	−0.237	−0.054	0.148	1.435
Locus4	−0.275	0.040	0.247	0.762
Locus5	−0.365	−0.194	0.125	1.745
Locus6	−0.166	0.117	0.243	0.780
Locus7	−0.309	−0.044	0.202	0.987
Locus8	−0.294	0.033	0.253	0.740
Locus9	−0.294	0.009	0.235	0.816
Locus10	−0.129	0.109	0.211	0.936
Locus11	−0.237	0.132	0.298	0.589
Locus12	−0.269	−0.052	0.171	1.212
Locus13	−0.413	−0.147	0.188	1.079
***Mean***	−0.274	0.005	0.220	0.961
***SE***	0.023	0.032	0.015	0.094

Fis, inbreeding coefficient within populations; Fit, total inbreeding coefficient; Fst, fixation index; Nm, number of migrants per generation ((1 − Fst)/(4 × Fst)); *SE*, standard error.

**Table 4:** The collection sites, GPS coordinates, and elevations from which the *P. armeniaca* accessions were collected during the expeditions in the years 2023–2024.

Number of Accessions	GPS Coordinates, Elevations, m	Place of Collection, Year, Population
23	N43°22′.417′—N43°22′.832′ E077°35′.351′—E077°35′.868′, 980–1046 m	Turgen Gorge, Enbekshikazakh District, 2023–2024, population 1
43	N43°17′.118′—N43°18′.456′ E079°37′.901′—E079°39′.901′, 1302–1691 m	Bolshoy Aksu Gorge, Uyghur District, 2023–2024, population 2/site 1
24	N43°18′.072′—N43°18′.702′ E079°30′.658′—E079°32′.314′, 1580–1692 m	Bolshoy Kyrgyzsay Gorge, Uyghur District, 2023–2024, population 2/site 2
16	N43°20′.784′—N43°20′.875′ E079°29′.827′—E079°29′.890′, 1234–1246 m	Foothills of Bolshoy Kyrgyzsay Gorge, Uyghur District, 2023–2024, population 2/site 3
5	N43°31′.652′—N43°31′.674′ E079°27′.353′—E079°27′.849′, 862–864 m	Chundzha village, Karadala Forestry, 2024, population 2/site 4

**Table 5:** List of barcoding genes used for Polymerase Chain Reaction (PCR) and sequencing of *P. armeniaca*.

Name of Primers	Primer Sequence 5′-3′	Primer Annealing Temperature	Reference
*MatK*_*390*-F	CGATCTATTCATTCAATATTTC	53 °C	[165]
*MatK_1326*-R	TCTAGCACACGAAAGTCGAAGT		
*trnHF*_*05*-F	CGCGCATGGTGGATTCACAATCC	58 °C	[166]
*psbA3f*-R	GTTATGCATGAACGTAATGCTC		
*ITS-Bel3*-F	GACGCTTCTCCAGACTACAAT	60 °C	[167]
*ITS-p5*-R	CCTTATCACTTAGAGGAAGGAG		
*rbcLa*-F	ATGTCACCACAAACAGAGACTAAAGC	62 °C	[168]
*rbcLr590*	AGTCCACCGCGTAGACATTCAT		

**Table 6:** List of primers used for SSR genotyping.

Primer Code	Name	Fluorescent Label	Subsequence	Reference
Pr-1	240001	FAM	cagtttgatttgtgtgcctctc	[171]
	240002		gatccaccctttgcataaaatc	
Pr-2	240003	FAM	gtgcccacttacctgttttagg	[171]
	240004		tcgacgatcagacttgctacag	
Pr-3	240005	VIC	ctgagtgatccatttgcagg	[172]
	240006		agggcatctagacctcattgtt	
Pr-4	240007	NED	ttaagagtttgtgatgggaacc	[172]
	240008		aagcataatttagcataaccaagc	
Pr-5	240009	FAM	tcctgcgtagaagaaggtagc	[172]
	240010		cgacataaagtccaaatggc	
Pr-6	240011	PET	aattgtacttgccaatgctatga	[172]
	240012		ctgccttctgctcacacc	
Pr-7	240013	FAM	tatattgttggcttcttgcatg	[172]
	240014		tgaaagtgaaacaatggaagc	
Pr-8	240015	NED	atgaggacgtgtctgaatgg	[172]
	240016		agccaaacccctcttatacg	
Pr-9	240017	VIC	aattaactccaacagctcca	[173]
	240018		atggttgcttaattcaatgg	
Pr-10	240019	FAM	caattagctagagagaattattg	[173]
	240020		gacaagaagcaagtagtttg	
Pr-11	240021	PET	tgaatattgttcctcaattc	[173]
	240022		ctctaggcaagagatgaga	
Pr-12	240023	VIC	tcagcaaactagaaacaaa	[173]
	240024		ccttgcaatctggttgatgtt	
Pr-13	240025	PET	tcggtttttaaaattccaaaa	[173]
	240026		gttacccttatttgcacccaaca	
Pr-15	240029	PET	agggaaagtttctgctgcac	[174]
	240030		gctgaagacgacgatgatga	

## Data Availability

The datasets presented in the study are either included in the article or in the Supplementary Material; further inquiries can be directed to the corresponding authors.

## Supplementary Materials

The following supporting information can be downloaded at: https://www.mdpi.com/article/10.3390/plants14152333/s1, Table S1: Distribution of qualitative morphological characteristics of *Prunus armeniaca*. Table S2: The GPS coordinates and elevations from which the 54 *P. armeniaca* accessions were collected for molecular analysis.
