# Supplementary material for: Geobotanical Study, DNA Barcoding, and Simple Sequence Repeat (SSR) Marker Analysis to Determine the Population Structure and Genetic Diversity of Rare and Endangered Prunus armeniaca L
Source: Plants (Basel). 2025 Jul 28;14(15):2333. doi: 10.3390/plants14152333 (PMC12349625; doi:10.3390/plants14152333)
Supplement: Supplementary file 1 [file plants-14-02333-s001.zip › plants-3740340-supplementary.pdf]

plants

MDPI

Supplementary material

Geobotanical Study, DNA barcoding and Simple Sequence Repeat (SSR) Marker Analysis to Determine the Population Structure and Genetic Diversity of Rare and Endangered *Prunus armeniaca* L.

Table S1. Distribution of qualitative morphological characteristics of *P. armeniaca*.

Number of population / Descriptors		Quantity	Number of accession / Percentage				
1 Population	PD	23	LPD (15/65.2)	APD (8/34.8)			
	CA	23	OD (2/8.7)	AV (13/56.5)	YF (3/13.0)	YNF (5/21.7)	
	HA	23	LA (3/13.0)	A (5/21.7)	T (15/65.2)		
	SAC	23	PCS (1/4.3)	VSC (8/34.8)	RSC (6/26.1)	HSF (1/4.3)	IC (7/30.4)
	DAC	23	D (6/26.1)	A (14/60.9)	SD (3/13.0)		
	F	0	NF (23/100)				
	SL	23	OLS (15/65.2)	RS (8/34.8)			
	LE	23	LLE (3/13.0)	SLE (17/74.0)	LSE (3/13.0)		
2 Population, first site	PD	43	APD (9/20.9)	HPD (34/79.1)			
	CA	43	AV (30/69.8)	YF (7/16.3)	YNF (6/14.0)		
	HA	43	LA (5/11.6)	A (19/44.2)	T (19/44.2)		
	SAC	43	PCS (15/34.9)	VSC (18/41.9)	SCS (1/2.3)	IC (9/20.9)	
	DAC	43	D (22/51.2)	A (17/39.5)	SD (4/9.3)		
	F	0	NF (43/100)				
	SL	43	OLS (7/16.3)	RS (35/81.4)	ELS (1/2.3)		
	LE	43	LLE (4/9.3)	SLE (21/48.8)	LSE (7/16.3)	SSE (9/21.9)	SE (2/4.7)
2 Population, second site	PD	24	LPD (2/8.3)	APD (16/66.7)	HPD (6/26.1)		
	CA	24	AD (11/45.8)	AV (9/37.5)	YF (3/12.5)	DA (1/4.2)	
	HA	24	A (10/41.7)	T (14/58.3)			
	SAC	24	PCS (1/4.7)	VSC (12/50.0)	HSF (7/29.2)	RSC (2/8.3)	IC (2/8.3)
	DAC	24	D (16/66.7)	A (8/33.3)			
	F	1	NF (23/95.8)	WF (1/100)			
	SL	24	OLS (2/8.3)	RS (11/45.8)	ELS (11/45.8)		
	LE	24	LLE (5/20.8)	SLE (11/45.8)	LSE (3/12.5)	SSE (5/20.0)	
2 Population, third site	PD	16	HPD (16/100)				
	CA	16	AV (7/43.8)	YF (9/56.3)			
	HA	16	A (15/93.8)	T (1/6.3)			
	SAC	16	PCS (10/63.0)	RSC (1/6.3)	IC (3/18.8)	VSC (1/6.3)	
	DAC	16	D (9/56.3)	A (6/37.5)	SD (1/6.3)		
	F	16	WF (1/6.3)	AFA (4/25.0)	VSC (11/68.8)		
	SL	16	OLS (6/40.0)	RS (10/60.0)			
	LE	16	SLE (16/100)				
2 Population, fourth site	PD	5	APD (5/100)				
	CA	5	AV (3/60.0)	YF (2/40.0)			
	HA	5	A (5/100)				
	SAC	5	RSC (1/20.0)	IC (4/80.0)			
	DAC	5	D (2/40.0)	A (3/60.0)			
	F	5	AFA (3/60.0)	VSC (2/40.0)			
	SL	5	OLS (2/40.0)	RS (1/20.0)	ELS (2/40.0)		
	LE	5	LLE (2/40.0)	SLE (3/60.0)			

* Descriptors: Condition of the accession (CA); Density of the accession crown (DAC); Fruiting (F); Height of the accession (HA); Leaf edge (LE); Population density (PD); Shape of the accession crown (SAC); Shape of the leaf (SL).
Morphological features: Average population density (APD); Adult diseased (AD); Abundant fruiting (AF); Average fruiting accession (AFA); Adult viable (AV); Dense (D); Dying accession (DA); Elliptic leaf shape (ELS); Heart-shape of the fruits (HSF); High population density (HPD); Irregular crown (IC); Low accession (LA); Lobed leaf edge (LLE); Low population density (LPD); Largely serrated leaf edge (LSE); Narrow (N); No fruiting (NF); Old decrepit (OD); Ovate leaf shape (OLS); Pyramidal crown shape (PCS); Round shape (RS); Round shape crown (RSC); Spreading crown shape (SCS); Sparse density (SD); Serrated leaf edge (SE); Small-lobed leaf edge (SLE); Small-serrated leaf edge (SSE); Tall (T); Vase shape crown (VSC); Weak fruiting (WF); Young fruitful (YF); Young non-fruitful (YNF).

Table S2. The GPS coordinates, and elevations from which the 54 *P. armeniaca* accessions were collected for molecular analysis

Identification of Accessions	Population	Name of the original population. Location	Longitude (E)	Latitude (N)	Elevation (m)
17	Pop 1	Population 2. Site 1. Gorge " Bolshoy Aksu"	E79°39'. 9,00''	N43°17'.1,19''	1307 м
18			E79°39'. 9,03''	N43°17'.1,20''	1304 м
19			E79°39'. 9,01''	N43°17'.1,18''	1302 м
21	Pop 2	Population 2. Site 1. Gorge "Bolshoe Aksu"	E79°37'. 9,26''	N43°17'.5,85''	1631 м
22			E79°37'. 9,22''	N43°17'.5,88''	1631 м
23			E79°37'. 9,09''	N43°17'.5,92''	1621 м
24			E79°37'. 9,18''	N43°17'.5,88''	1634 м
25			E79°37'. 9,20''	N43°17'.5,91''	1643 м
26			E79°37'. 9,13''	N43°17'.5,95''	1641 м
27			E79°37'. 9,13''	N43°17'.6,01''	1647 м
28			E79°37'. 9,15''	N43°17'.5,97''	1645 м
29			E79°37'. 9,12''	N43°17'.6,07''	1658 м
30			E79°37'. 9,21''	N43°17'.5,98''	1751 м
31			E79°37'. 9,01''	N43°17'.5,99''	1691 м
32			E79°37'. 9,11''	N43°17'.5,86''	1679 м
33			E79°37'. 9,07''	N43°17'.5,92''	1652 м
34			E79°37'. 9,01''	N43°17'.5,96''	1640 м
35			E79°37'. 9,08''	N43°17'.5,99''	1641 м
36			E79°37'. 9,27''	N43°17'.6,06''	1657 м
37			E79°37'. 9,16''	N43°17'.6,01''	1643 м
38			E79°37'. 9,18''	N43°17'.6,02''	1645 м
20	Pop 3	Population 2. Site 1. Gorge " Bolshoy Aksu"	E79°37'. 9,31''	N43°17'.7,57''	1638 м
39	Pop 4	Population 2. Site 1. Gorge " Bolshoy Aksu"	E79°37'. 9,52''	N43°17'.9,73''	1599 м
40			E79°37'. 9,51''	N43°17'.9,27''	1593 м
41			E79°37'. 9,40''	N43°17'.9,16''	1588 м
42			E79°37'. 9,38''	N43°17'.9,11''	1587 м
43			E79°37'. 9,29''	N43°17'.9,29''	1599 м
44			E79°37'. 9,31''	N43°17'.9,29''	1580 м
45	Pop 5	Population 2. Site 2. Gorge " Bolshoy Kyrgyzsai"	E79°32'. 2,78''	N43°18'.0,73''	1688 м
46			E79°32'. 2,76''	N43°18'.0,80''	1684 м
47			E79°32'. 2,80''	N43°18'.0,83''	1689 м
48			E79°32'. 2,89''	N43°18'.0,88''	1692 м
49			E79°32'. 2,89''	N43°18'.0,90''	1689 м
50	Pop 6	Population 2. Site 2. Gorge " Bolshoy Kyrgyzsai"	E79°32'. 2,63''	N43°13'.0,72''	1676 м
51	Pop 7	Population 2. Site 2. Gorge "Bolshoy Kyrgyzsai"	E79°32'. 2,56''	N43°18'.6,97''	1588 м
52			E79°32'. 2,72''	N43°18'.6,93''	1580 м
53			E79°32'. 2,78''	N43°18'.7,01''	1586 м

Table S2. *Cont.*

1	Pop 8	Population 1. Gorge "Turgen"	E77°35'. 3,75''	N43°22'.8,18''	1021 м
2			E77°35'. 3,59''	N43°22'.8,25''	1022 м
3			E77°35'. 3,55''	N43°22'.8,24''	1026 м
4			E77°35'. 3,65''	N43°22'.8,23''	1031 м
5			E77°35'. 3,59''	N43°22'.8,25''	1022 м
6			E77°35'. 3,65''	N43°22'.8,31''	1033 м
10	Pop 9	Population 1. Gorge "Turgen"	E77°35'. 5,43''	N43°22'.6,70''	1034 м
11			E77°35'. 5,37''	N43°22'.6,67''	1042 м
12			E77°35'. 5,29''	N43°22'.6,71''	1035 м
13	Pop 10	Population 1. Gorge "Turgen"	E77°35'. 8,61''	N43°22'.4,51''	1046 м
14			E77°35'. 8,63''	N43°22'.4,45''	1044 м
15			E77°35'. 8,67''	N43°22'.4,44''	1000 м
16			E77°35'. 8,68''	N43°22'.4,42''	1045 м
55	Pop 11	Population 2. Site 2. Gorge " Bolshoy Kyrgyzsai"	E79°32'. 3,05''	N43°18'.7,02''	1595 м
56			E79°32'. 3,14''	N43°18'.6,98''	1592 м
57			E79°32'. 2,92''	N43°18'.6,92''	1592 м
58			E79°32'. 2,81''	N43°18'.6,88''	1583 м
